# Language can shape the perception of oriented objects

**DOI:** 10.1038/s41598-020-65455-6

**Published:** 2020-05-21

**Authors:** Eduardo Navarrete, Michele Miozzo, Francesca Peressotti

**Affiliations:** 1 Dipartimento di Psicologia dello Sviluppo e della Socializzazione, Università di Padova, Padova, 35137 Italy; 2 Columbia University, New York, 10027 USA

**Keywords:** Cognitive neuroscience, Human behaviour

## Abstract

Seeing an object is a natural source for learning about the object’s configuration. We show that language can also shape our knowledge about visual objects. We investigated sign language that enables deaf individuals to communicate through hand movements with as much expressive power as any other natural language. A few signs represent objects in a specific orientation. Sign-language users (signers) recognized visual objects faster when oriented as in the sign, and this match in orientation elicited specific brain responses in signers, as measured by event-related potentials (ERPs). Further analyses suggested that signers’ responsiveness to object orientation derived from changes in the visual object representations induced by the signs. Our results also show that language facilitates discrimination between objects of the same kind (e.g., *different cars*), an effect never reported before with spoken languages. By focusing on sign language we could better characterize the impact of language (a uniquely human ability) on object visual processing.

## Introduction

Much of what humans know about visual objects is learned implicitly by seeing the objects and is shaped by their sensibility for recognizing object features that are encountered more commonly and predictably. The brain mechanisms supporting this type of learning are largely shared with other animal species^1^. But humans evolved the unique ability to create and manipulate symbols that represent visual objects and are understood to stand for the objects themselves. Do these symbols provide humans with an additional source for learning implicitly about visual objects? Could a symbol-based form of knowledge enrich what is learned directly from visual objects? The spoken languages that emerged from human evolution are built on words, symbols providing humans a powerful cognitive tool for identifying visual objects. In light of their representative capacity, words would stand as primary candidates for contributing to knowledge about visual objects. Words have consistently been found to modulate visual discrimination, affecting the initial stages of color and object identification^2–4^. These word effects have been linked to words’ primary role in defining conceptual categories^3,5^. As category labels, words participate in strengthening conceptual boundaries and grouping similar objects. Words can thus down-regulate even early processes of visual object recognition by providing diagnostic information for disentangling different sources of neural activation.

Distinguishing individual exemplars requires identifying variations in the features that set apart each visual object within its category. It is mainly by linking words into phrases (*“The red car”*; *“The long car”*) that speakers can mark these distinctions. Individual words are in this respect quite ineffectual given their lack of reference to specific visual features – nothing in the form of the word *car* tells us of the shape, orientation or size of a given car. This limitation is not shared by signs, the equivalent of words in the sign languages that deaf people use to communicate. As signing is grounded in shaping and moving the hands and in changes of facial expression^6–8^, signs can display specific visual features of the corresponding objects, including shape, orientation, and size^9–11^. We turned to signs to investigate if linguistic symbols provide the kind of knowledge needed to discriminate between different instances of an object, which relates to object features such as orientation, color, size, and shape.

Studies have shown that knowing a sign language improved performance in a variety of visuo-spatial tasks, which in turn led to the realization that signing affects spatial cognition^12^, response mapping^13^, visual mental imagery^14^, and face processing^15^. The effects of sign language have been associated with specific characteristics of the language, including the use of hand configuration, location, and facial expression to mark grammatical and lexical distinctions. For example, signers’ ability to rotate visual images was related to the use of sign language to describe spatial relations, which signers define from a different perspective than the viewers who try to understand them^14^. The effects of sign language documented in prior studies were general in nature, stemming from core features of the language and implicating visual processes that, like mental imagery, apply to a wide range of stimuli. We explored if the effects of sign language are more specific, involving distinctive object features that affect the appearance and identity of objects from a given category. We focused on object orientation. Orientation is a diagnostic feature of object variation – changes in object orientation are associated with variations in object’s position and motion or reflect interactions with other objects (e.g., hitting). Changes in orientation also help to recognize if there are multiple instances of the same objects. By investigating orientation, we could examine effects related to the recognition of specific objects in a given category.

**Figure 1:**
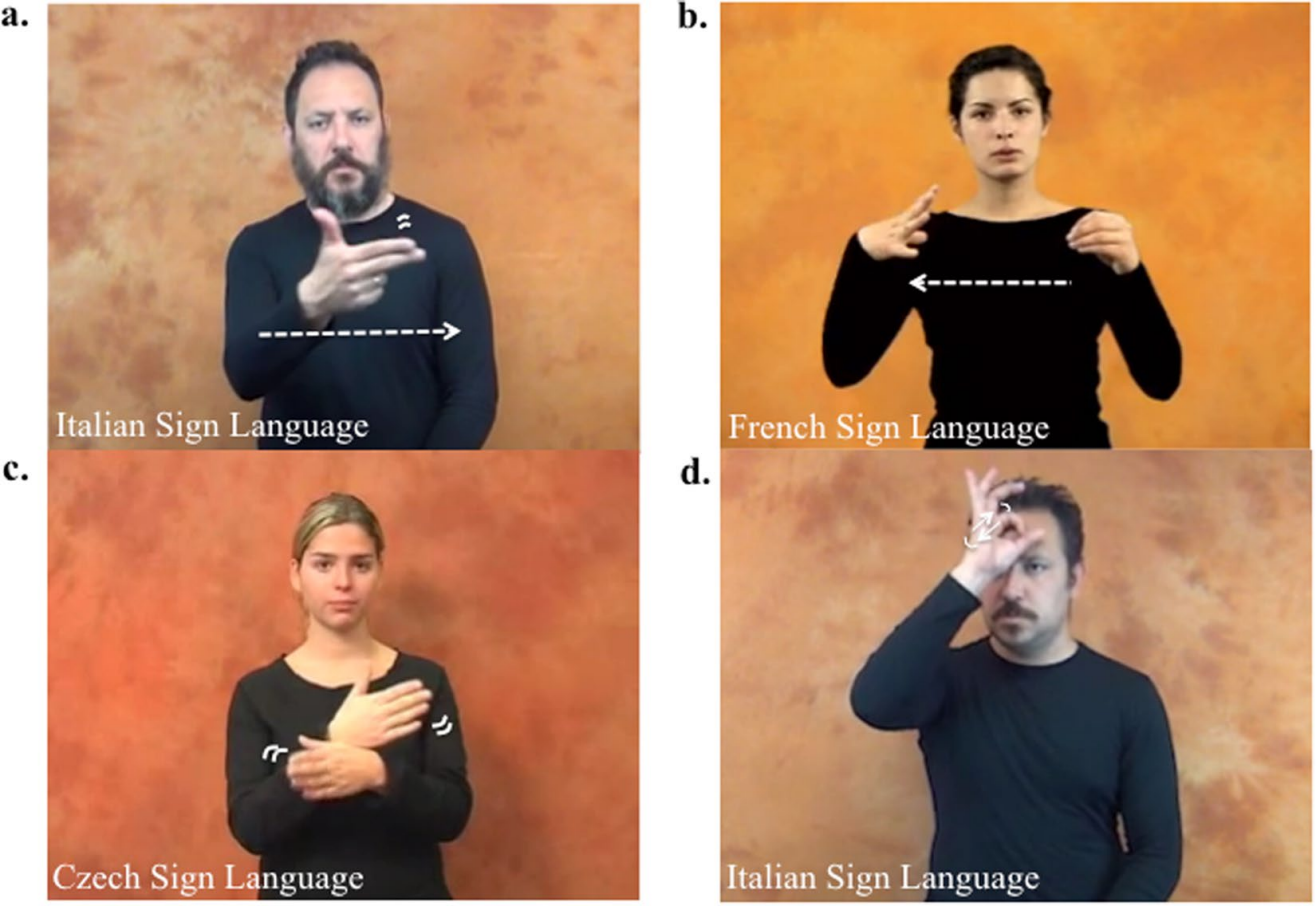
Examples of the signs *fish* (Panel a,c) and *zucchini* (Panel b,d) in different sign languages. *Fish* is represented in a specific orientation in the Italian Sign Language (Panel a) but not in the Czech Sign Language (Panel c), while *zucchini* is represented in a specific orientation in the French Sign Language (Panel b) but not in the Italian Sign Language (Panel d). The pictures of the signs were taken from https://www.spreadthesign.com/it.

**Figure 2:**
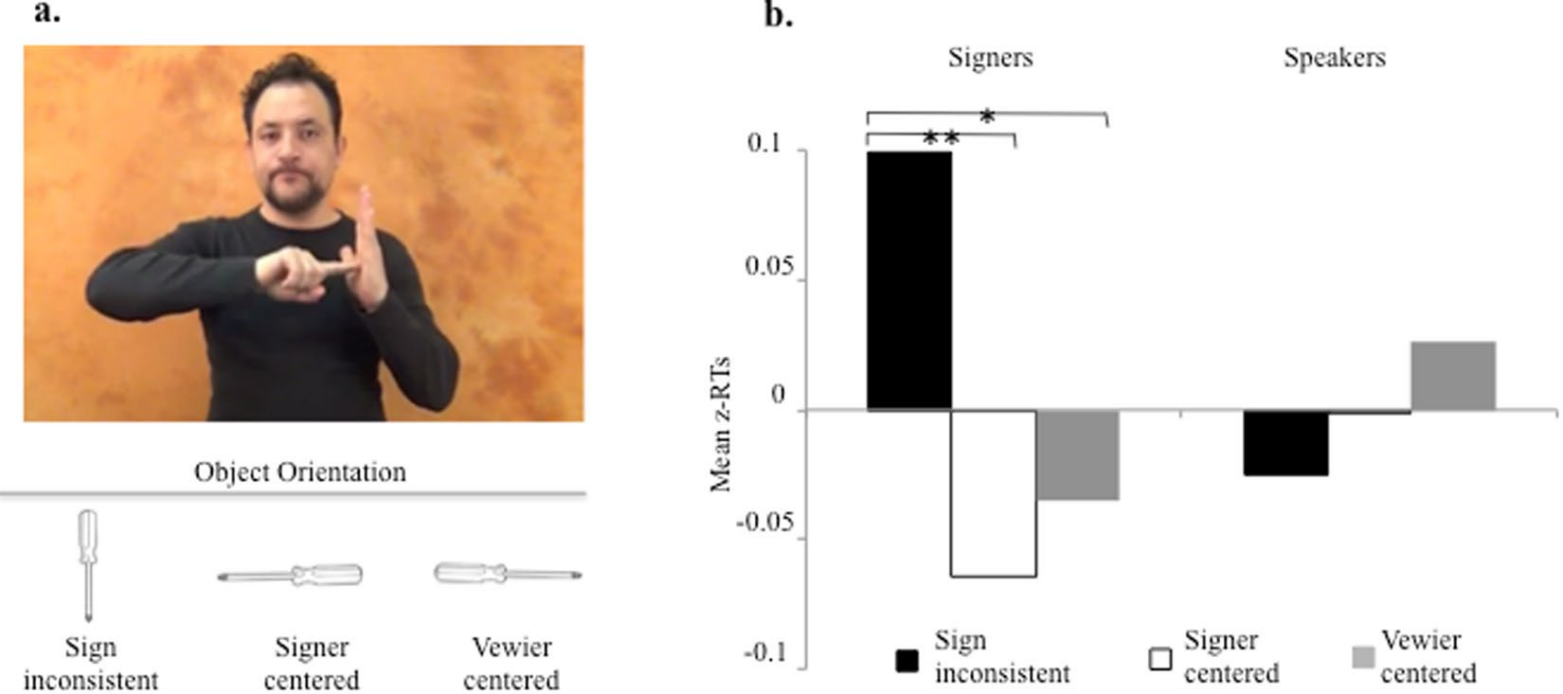
Panel (**a**) Each of the objects tested in Experiment 1 appeared in three orientations determined with respect to the orientation in which the objects are displayed in the signs. The example illustrated here shows the pictures used for screwdriver and the sign *screwdriver* in Italian Sign Language (https://www.spreadthesign.com/it) in Experiment 1. When facing the signer, the orientation in which the sign depicts the object is from the perspective of the person viewing the signer (viewer-centered). Such orientation is mirror-reversed when the sign is viewed from the signer’s perspective (signer-centered). Panel (**b**) Response times (z-score transformed) recorded for signers and speakers when the words matched the objects (Yes responses). Signers responded faster when the objects were oriented as in the signs (either according to a viewer-centered perspective or a signer centered perspective). Such differences were not found with speakers.

As illustrated in Fig. 1, signs from multiple languages iconically display objects in a specific orientation, as with the sign *fish*, showing fish with the head pointing toward the signer’s left side. We took advantage of this distinct aspect of signs to examine if linguistic symbols enable within-category discriminations. In Experiment 1, signers and speakers decided if words matched the pictured objects. Object orientation either aligned with or differed from the object orientation embedded in the sign (Fig. 2a). If signs shape expectations about object orientation, only signers’ responses should be affected by whether signs displayed objects in a particular orientation. Previous studies reported faster responses in a sign-picture matching task when the visual features of the objects shown in the pictures were iconically represented in the signs^16^. In contrast with previous studies, our task did not involve sign presentation and was not based on the perceived similarity of visual stimuli.

In Experiment 2 orientation was similarly varied, where we recorded the brain’s event-related potentials (ERPs) elicited by changes in object orientation. We anticipated that only signers’ ERPs would show brain correlates associated with the explicit encoding of orientation by the sign.

## Experiment 1

We selected 28 objects, each represented by a sign displaying the object in a specific orientation. The example of screwdriver is presented in Fig. 2. As illustrated in Fig. 2, the orientation in which an object appears in the sign varies between signer and viewer perspectives. As we did not know which perspective was critical, we tested both of them. Each object appeared in two sign-consistent orientations (signer- and viewer-centered) and a sign-inconsistent orientation (Fig. 2a). To test deaf signers (n = 24) and speakers (n = 24) with the same task, we presented written words. Thus, participants decided as fast as possible whether or not the written word matched the picture presented next. In half the trials the word and picture matched (Yes responses), in the other half they did not (No responses). We expect picture orientation to affect Yes responses for signers but not for speakers, who are unaware of sign language. We analyzed the effects of the predictors Orientation (signer-centered vs. viewer-centered vs. signer-inconsistent) and Group (signers vs. speakers) on the speed of Yes responses.

The results are displayed in Fig. 2b. Statistical analyses showed no effects of Orientation or Group (signers vs. speakers), ps > 0.18. Critically, the interaction between these two variables was significant, χ^2^ = 7.43, p = 0.024. Pairwise comparisons (fdr corrected) indicated that deaf participants responded faster to the signer-centered orientation *and* the viewer-centered orientation compared to the sign-inconsistent orientation, z = 2.97, p = 0.008 and z = 2.50, p = 0.018, respectively. With speakers these comparisons failed to reach the significance level, ps > 0.72. In essence, signers demonstrated a distinct responsiveness to orientation traceable to their knowledge of signs.

## Experiment 2

**Figure 3:**
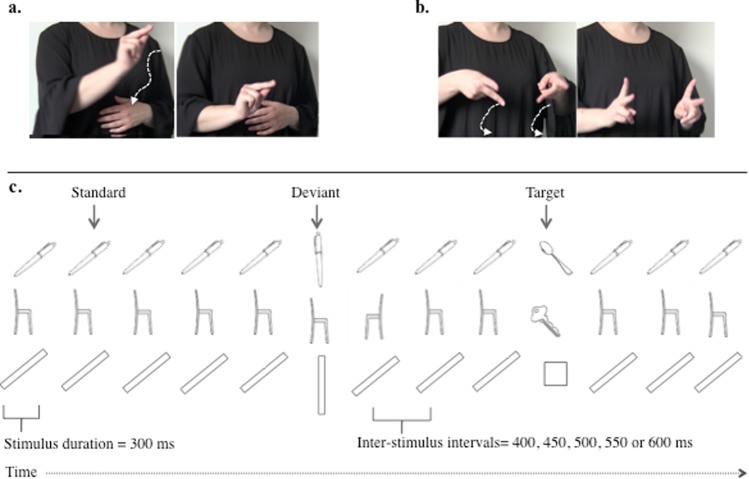
Illustration of the signs *pen* (Panel a) and *chair* (Panel b) in Italian Sign Language. Two snapshots were taken for each sign – one at the beginning, the other at the end of sign production. Panel (**c**) Sequence of stimuli of the three-stimulus oddball task used in Experiment 2 (pictures used available at https://osf.io/y4pb9/. Stimuli appeared in rapid succession, one at a time. Each row presents the stimuli tested in one of the experimental conditions. Participants responded only to the targets. The other stimuli in the sequence (standard and deviant) represented the same object in different orientations. Analyses focused on the difference between standard and deviant trials. In each sequence, the standard appeared with a far higher probability (0.80) than the deviant (0.15) and the target (0.05). The orientation of pen was either consistent with the orientation of pen in the sign *pen* from a signer perspective, or differed from it. The orientations of bar were the same as pen. The orientations of chair had no correspondence with the sign chair and created mirror reversed images of the pictured chair. Note that for each object a given orientation appeared as standard in a block and as deviant in another block.

Using a semantic judgment task, Meade *et al*.^17^ showed automatic sign activation in bilingual signers during word reading. Experiment 2 aimed to investigate the orientation effect without presenting words. We used ERPs to shed light on the brain underpinnings of the orientation effect found in the previous experiment, specifically to determine whether it involved object perceptual processing or response-related mechanisms. The ERP technique offers the unique opportunity to investigate the time course of cognitive processes even in trials in which no response is required. In Experiment 2 we measured brain’s event-related potentials (ERPs) elicited by orientation change in a three-stimuli oddball task. All the stimuli were object drawings. The task required responding to an infrequent target shown in a background of two other objects, one occurring frequently (standard) the other rarely (deviant). No responses are required to standard and deviant stimuli (Fig. 3). Deviants typically elicit larger positive amplitude of the electric signal compared to standards^18–22^. Furthermore, amplitude differences were found between standards and deviants varying in orientation, a result proving the paradigm’s sensitivity to object orientation^23^. In Experiment 2, the picture of a pen appeared as standard and deviant with an orientation that either aligned with the orientation encoded in the sign *pen*, from the signer perspectives, or with an orientation that differed from it (Fig. 3). In line with the results of Experiment 1, the orientation of pen was expected to elicit different responses in signers and speakers. To control that the effects found with signers for pen stemmed from the orientation in which pen is displayed in the sign, two additional conditions were included in Experiment 2. We presented the picture of a chair and a bar similar in shape to pen. Both stimuli were presented as standards and deviants, varying their orientation (Fig. 3). Since the sign *chair* does not encode object orientation, we expected the effects found with pen not to replicate with chair. Bar was shown in the same orientation as pen. We expected bar not to elicit orientation effects analogous to pen.

The oddball task involves discriminating if information about the incoming stimulus matches the information previously acquired about the standard^18–23^. This process is indexed by the deviancy effect, which arises over the posterior regions around 150 ms after stimulus onset, surfacing later at 250–500 ms as a positive amplitude increase over the midline electrodes, the typical configuration of P300 wave. When different colors or objects were shown as standard and deviant^2,3^, larger deviancy effects appeared at 150–200 ms in the languages where distinct words existed for the stimuli – e.g., in English, where the *cup*/*mug* distinction is lexically marked, but not in Spanish where the same word is used for both objects. As category labels, words thus appear to affect the earlier processes supporting initial, crude object discriminations^3,5^. The within-category discrimination required in the present study to distinguishing between standards and deviants, would depend on more refined visual object representations that are accessed at later stages or processing^18^. We therefore expect that the later P300 component would index the effects of signs on orientation.

**Figure 4:**
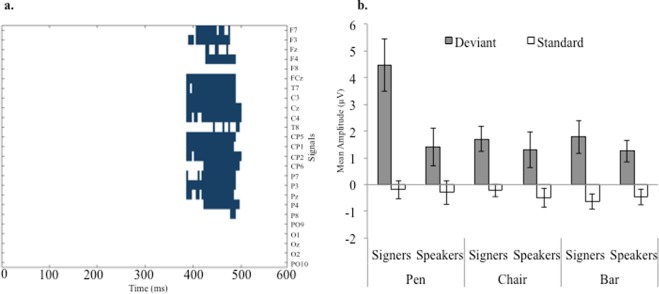
Panel (a) Cluster-based permutative analysis were conducted to determine the electrode clusters and the time window in which the deviancy effect (deviant *minus* standard) occurred. Regions in which the deviancy effect was significantly larger (p < 0.05) for signers than speakers are shown in blue. A significant effect was found only for pen, which concentrated in the P300 temporal region. (The non-significant results from chair and bar are not shown). Panel (b) Mean amplitudes elicited by standards and deviants over the Pz electrode between 250 and 550 ms post stimulus-onset (P300). For each type of stimuli (pen, chair, and bar), orientation change elicited a significant deviancy effects (i.e., larger positive amplitudes for deviants). However, only for pen the deviancy effect was a significantly larger for signers than speakers. Error bars indicate standard errors.

**Figure 5:**
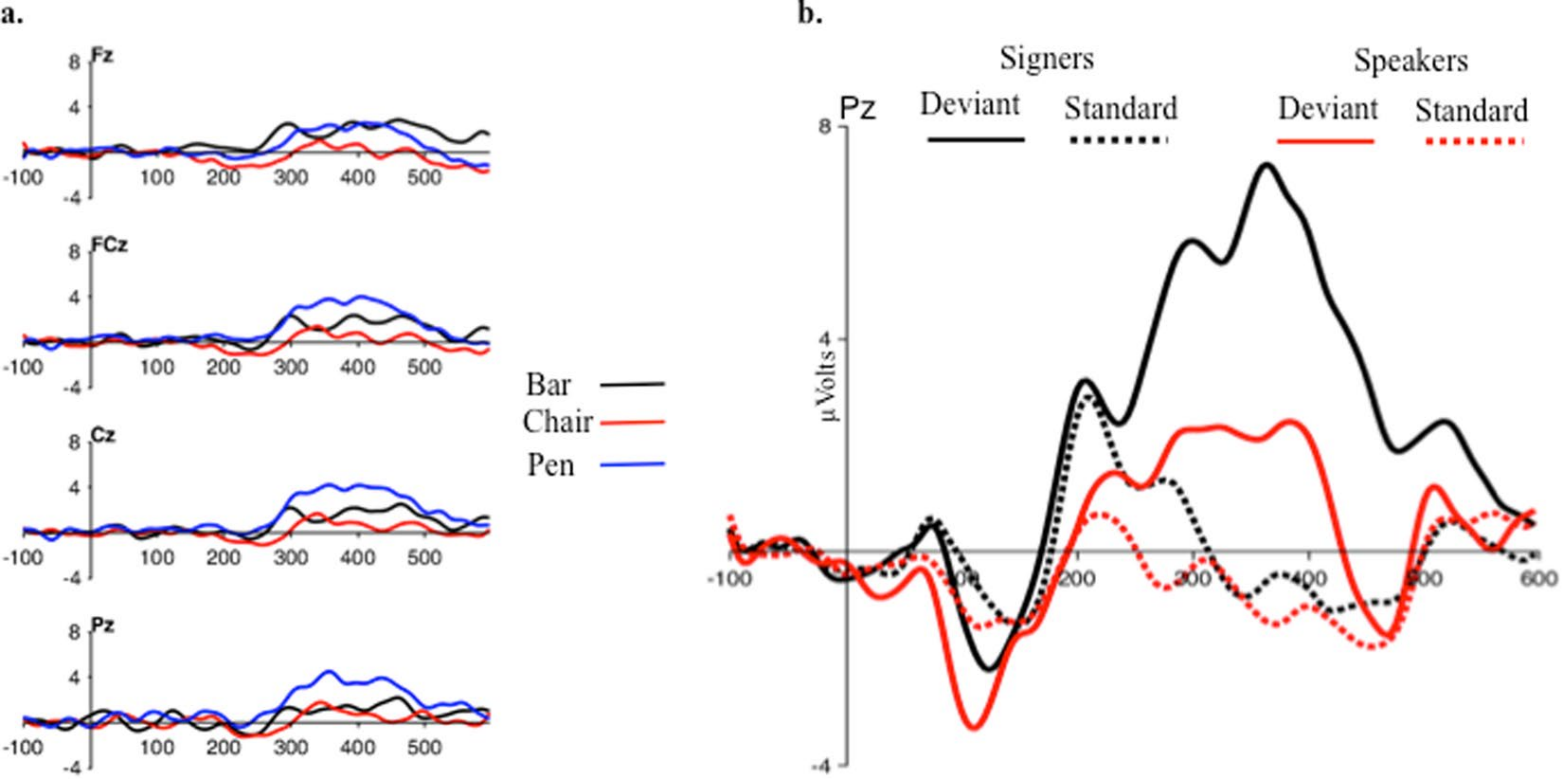
Panel (a) Waveforms generated by subtracting speakers’ deviancy effects from signers’ deviancy effects. Deviancy effects indicate larger positive amplitudes for deviants relative to standards. Waveforms correspond to differences obtained with pen, chair, and bar in four central electrodes (Fz, FCz, Cz, and Pz). In the 250–550 ms interval, a larger deviancy effect appeared for signers with pen, especially in posterior electrodes (Pz). Panel (b) ERPs elicited by the standard and deviant pen in the Pz electrode (signers: black lines; speakers: red lines). Only the deviant pen elicited larger amplitudes in signers than in speakers.

In a first data-driven analysis, we compared the deviancy effect elicited in signers and speakers by each object (pen, chair, bar) from stimulus onset up to 600 ms, using non-parametric cluster-based permutations. A larger deviancy effect for signers was found only for pen between 380 and 500 ms after stimulus presentation over central/parietal electrodes, p = 0.025, (Fig. 4a). These results aligned with our predictions of group differences limited to pen and the P300 component. Next, we carried out planned analyses on P300 amplitude, examining the effects of the variables Deviancy (Standard vs. Deviant), Group (Signers vs. Speakers), and Stimulus Type (Pen vs. Chair vs. Bar) on the responses recorded at 250–550 ms over the Pz electrode. The significant triple interaction revealed that the deviancy effect varied between groups and across stimuli, F(2, 36) = 5.47, p = 0.008, ηG^2^ = 0.036 (Fig. 4b). Additional analyses were carried out to elucidate the nature of the interaction. First, when Deviancy and Stimulus Type were examined separately for signers and speakers, the interaction was significant only for signers, F(2, 18) = 7.44, p = 0.004, ηG^2^ = 0.116; for speakers, F < 1. Second, Deviancy and Group were examined separately for each type of stimuli; a significant interaction was found only for pen, F(1, 18) = 7.72, p = 0.012, ηG^2^ = 0.121; other Fs <1 (Fig. 5a). These additional analyses showed that in P300, differences between signers and speakers are confined to pen. A final analysis aimed to determine if the effects related to pen were driven by the standard or the deviant. The variables Group and Distractor Type were examined separately for standard and deviant. The interaction was significant for the deviant and not for the standard, F(2, 36) = 5.79, p = 0.007, ηG^2^ = 0.087; F < 1. The latter finding revealed that group differences were elicited only by the deviant (Fig. 5b).

## General Discussion

The orientation effects we found were specific, first for appearing with signers and not speakers (Exp. 1 and 2), and second for involving only objects whose signs visually encode object orientation (Exp. 2). This specificity was expected under the hypothesis that signers are especially responsive to objects oriented as in the signs. This hypothesis also anticipated – as we found – that the orientation effects would engage the brain mechanisms of object processing indexed by P300.

Importantly, the specificity of our findings ruled out that brain processes other than those supporting visual object recognition underlay the sign effect. Action processing would be a plausible candidate considering the relevance of object orientation for actions toward the object (e.g., grasping) along with findings showing that even viewing an object picture could trigger the planning of an action^24–27^. However, an explanation in terms of action processing is inconsistent with the specificity of our findings. As signers and speakers have had reasonably similar experiences in using objects, this explanation anticipates their responses not to differ. Another plausible candidate is mental rotation, possibly needed in our tasks to align the stimuli to a canonical orientation. Notably, differences between signers and speakers could have stemmed from signers’ skillfulness in mental rotation^14^. However, an explanation in terms of mental rotation predicts group differences with all the objects we tested, not just those with signs embedding object orientation.

In what way would signs affect object orientation? Under a lexical hypothesis, it would result from the implicit and automatic activation of information concerning the sign^17^. This hypothesis aligns with accounts proposing that higher levels of processing influence lower ones, such as the interactive-activation models^28,29^. According to this view, bottom up sensory processes and prior knowledge are jointly used and integrated in order to determine the contents of the perceptual experience^30^. From this perspective, the visual object would activate information about the shape, location, orientation, and movement involved with the sign. The effects found in our tasks would then depend on whether or not objects aligned with sign orientation. Thus, for example, matching responses would be faster in Experiment 1 when the orientation of the object and the sign coincided, and slower otherwise. An a-posteriori test of the lexical hypothesis was conducted on the basis of prior evidence from picture signing. ERPs correlates of lexical activation were found when comparing iconic and non-iconic signs^31^. Iconic signs, which reproduce distinguishing features of objects, elicited more positive amplitudes than non-iconic signs in posterior sites at around 100 ms post stimulus-onset (P100). We inspected if similar differences arose in Experiment 2 between pen (iconic) and chair (non-iconic) in posterior-occipital regions at 70–140 ms (see Methods for details). We found no iconicity effects, Fs < 1, disconfirming the lexical hypothesis. It should be noted, however, that differences in the paradigms used (oddball paradigm Vs. to standard picture naming) could also account for the lack of iconicity effect in our study and therefore the hypothesis of automatic and implicit sign activation cannot be fully rejected.

Alternatively, signs could affect object representations by changing feature weights, a cognitive correlate of feature distinctiveness shaping expectations. Their larger weights make salient features more expected, so that their detection becomes crucial in object recognition. With orientation, the weight increase would make certain orientations more canonical and therefore more expected. Embodying object orientation into the sign could also strengthen the saliency of certain orientations. Therefore, as we expect to see a bottle upright or a monitor facing us^32^, signers would expect objects to align as in the signs. Individual signs would thus warp visual perception by sensitizing to the object features they transparently encode.

Our findings revealed that by affecting the processing of specific visual features and distinct objects, signs contribute to processes enabling the discrimination of different instances of an object. Signs re-map the boundaries of the language-vision interface drawn upon spoken language. While prior evidence showed that words affect categorical discriminations and modulate earlier neural processes in visual object recognition^5,33,34^, our results from signs suggest language effects in the later stages of object processing responsible for detecting object changes. Disclosing more fully the language’s potential to shape visual cognition, signs point to other types of symbols incorporating object features that may also affect within-category discriminations. One example is represented by spoken words sounding like noises, calls, object sounds, voice features or/musical elements, as with the English words *bang*, *quack*, *click*, *grunt* or *bong*, respectively. These types of words could influence acoustic discrimination. If the reason for the sign effects documented in our experiments is that the complexity of object processing makes any processing aids valuable, human brains should also exploit the opportunities offered by any type of symbol embodying object features^35^. In fact, permeability to symbols would be a necessity, and taking advantage of the opportunities offered by symbols an adaptive choice.

## Methods

### Experiment 1

#### Participants

Required sample was estimated using the software GPower 3.1^36^, based on the effect size obtained by Thompson *et al*.^11^. Precisely, Signer participants (n = 24, 13 males; age: mean = 31.3, range = 18–53, SD = 10.1) presented with profound bilateral hearing loss (>90 dB). 16 participants were exposed to Italian Sign Language [*Lingua Italiana dei segni*, LIS] from birth, acquiring it from deaf parents, and used it as their primary and preferred form of communication; 8 participants acquired LIS between 3–12 years old (mean = 8, SD = 3.3) and had at least 10 years of exposure to LIS (mean = 21.6, range = 10–42, SD = 9.9). Signers were fluent in LIS (mean rate = 6.6, range = 4–7, SD = 0.8, on a 7-point scale from 1-no proficiency, to 7-native signer). Hearing native Italian speakers with no knowledge of sign language were recruited for the control group (n = 24, 10 males; mean age = 22.3, range = 20–29, SD = 2.19). All participants were right-handed and had normal or corrected-to-normal vision. Individuals participated in Experiment 1 and 2 voluntarily and provided informed consent. The study was approved by the Ethical Committee for Psychological Research of the University of Padova (Number 2455). Informed consent was obtained from all participants. Experiments have been performed in accordance with relevant guidelines and regulations.

#### Materials and procedure

We tested 28 pictured objects (stimulus list in Appendix; pictures available at https://osf.io/y4pb9/). Each pictured object was shown in black-and-white in three different orientations (Fig. 2). In two of these orientations, objects were oriented as in the corresponding signs viewed from the perspective of the signer (signer-centered) or the viewer’s (viewer-centered). Specular images of the same object were created to show these orientations. In a third orientation (sign-inconsistent), objects were rotated ~90° from the signer-centered perspective. An object appeared in a specific orientation twice, once preceded by the object name (Yes response) and once by the name of another object tested in Experiment 1 (No response).

Each experimental trial started with a fixation point displayed for 300 ms and replaced by a written word (Times Roman font, 28 points) presented for 2 s; next, a blank screen appeared for a randomly selected duration of 200, 400, or 800 ms, followed by a picture that stayed in view until the response or up to 3 s. The next trial started automatically after 1 or 2 s. Words and pictures appeared in the center of the computer screen. Participants decided as fast and accurately as possible if words and pictures matched by pressing the s key of the keyboard with the left hand for Yes responses (“Sì” in Italian), and the n key with the right hand for No responses (“No” in Italian). Participants were first familiarized with the task with a novel set of 4 warm-up words and pictures for 8 trials (4 Yes responses, 4 No responses). The 168 trials were presented in a pseudo-randomized order, so that pictures and words were not repeated in the next trial, and the same response did not occur in more than four consecutive trials. There was a short pause after 42 trials. Response times (RTs) were measured from picture onset. Stimuli presentation and response recording were controlled by E-Prime 2.0 (Psychology Software Tools, Inc., Sharpsburg, Pittsburgh, PA).

#### Analyses

RT-outliers (2.5% of the responses) were removed using the procedure recommended by Van Selst and Jolicoueur^37^. Incorrect responses occurred rarely with both signers (2.2%) and speakers (3.1%) and were not analyzed. Analyses were performed on Yes responses. Given the slower reading-times typically experienced by deaf readers^38,39^, raw scores of the RTs of the two groups were transformed into z scores to correct for differences in processing speed and variability across groups of participants^40^. Z scores were analyzed through linear mixed effects models and accuracies through generalized linear mixed effects models using the lm4^41^ package in R software^42^. Predictors were Participants (signers, speakers) and Orientation (signer-centered, viewer-centered, sign-inconsistent), and we included by-item and by-participant random effects.

### Experiment 2

#### Participants

Based on Azizian *et al*.^43^ who measured the P300 effect in an oddball paradigm with stimuli from the same category and established the expected effect-size, we opted for a sample of 20 participants overall. This choice took into account (a) the difficulty in recruiting right-handed profound deaf signers, aged below 40 years, without cochlear implants, who were exposed to sign language from birth or early childhood, familiar with the same variant of Italian Sign Language used in the city of Padua, agreeing to EEG measurement and (b) the fact that we examined a robust psychophysiological effect. Signers (n = 10, 4 males; age: mean = 21.1, range = 18–32, SD = 4.3) were congenital deaf with profound bilateral hearing loss (>90 dB) who were exposed to LIS from birth, acquired it from deaf parents and used it as their primary and preferred form of communication. Speakers were hearing native Italian speakers with no knowledge of any sign language (n = 10, 4 males, age: mean = 22.9, range = 19–29, SD = 2.7). None of the participants participated in Experiment 1. All participants were right-handed and had normal or corrected-to-normal vision. The study was approved by the Ethical Committee for Psychological Research of the University of Padova (Number 2455). Informed consent was obtained from all participants. Experiments have been performed in accordance with relevant guidelines and regulations.

#### Materials and procedure

We selected three sets of visual stimuli, each comprising one target, one standard, and one deviant. In each set, standard and deviant showed the same object, but in different orientations (Fig. 3; stimuli are available at https://osf.io/y4pb9/). The stimuli were manipulable objects in two of the sets (spoon and pen; key and chair), and geometrical shapes in the other set (square and bar). Each stimulus was presented in three orientations. Pen was shown in two orientations. Bar was oriented as pen. Chair was rotated either toward the right or the left, the mirror-image rotation that signers perform especially skillfully^14^. Objects were displayed as black-and-white drawings on a white background, whereas the geometrical shapes were shown in white on a black background. Stimuli subtended approximately 8° of visual angle and were presented in the center of a CRT monitor. Stimuli appeared with different probabilities: 0.80 for standards, 0.15 for deviants, and 0.05 for targets (see Fig. 3). The experiment comprised six blocks of 480 trials each. Blocks were presented in counterbalanced order. The same object appeared as standard and deviant in two consecutive blocks; the object orientation that was assigned to standard and deviant was reversed between these blocks.

Stimuli appeared for 300 ms, separated by inter-stimulus intervals that varied randomly in duration (400, 450, 500, 550 or 600 ms) but were used evenly. Stimuli presentation followed a quasi-random order in which targets and deviants never appeared in consecutive trials and the next deviant was never shown until at least three standards appeared in a row. Instructions, which were written in Italian and presented on the computer screen at the beginning of each block, informed of the next target (key, spoon, or square). Responses to targets were made by pressing the space bar of the keyboard. E-Prime 2.0 (Psychology Software Tools, Inc., Sharpsburg, Pittsburgh, PA) was used for stimuli presentation and response recording. Participants were seated comfortably at 60 cm from the computer screen in a noise- and light-attenuated room. The experimental session lasted about 1.5 hours.

#### EEG recording and data analysis

EEG activity was recorded continually using 32 Ag-AgCl electrodes that were referenced to the left earlobe and mounted on an elastic cap according to the extended 10–20 system. Horizontal EOG (HEOG) was recorded bipolarly from electrodes positioned lateral to the outer canthi of both eyes. Vertical EOG (VEOG) was recorded bipolarly from two electrodes, one above (Fp1) and one below the left eye. EEG, HEOG, and VEOG signals were amplified and filtered using a bandpass of 0.01–80 Hz and digitized at a sampling rate of 250 Hz. Impedance at each electrode site was maintained below 5 kΩ.

EEG recordings were processed offline using the MATLAB toolbox EEGLAB^44^. EEG was re-referenced off-line to the average of the left and right earlobes, and low- and high-pass filtered (30-0.1 Hz). The EEG was segmented into 700 ms epochs starting 100 ms prior to the onset of the stimuli. Epochs at each electrode site were baseline-corrected with reference to mean activity within the 100 ms preceding stimuli presentation. Bad channels were interpolated with the surrounding electrodes. With 3 participants one bad channel was interpoled. Epochs were subjected to independent component analysis (ICA^45^) to detect highly characteristic artefacts (e.g., eye blinks). Artefactual components were discarded, along with epochs that contained excessive noise or drift (signals exceeding ± 100 μV at any electrode). Epochs were averaged for each participant across the two conditions of interest (standards and deviants). Each average was based on data from at least 90 trials.

A data-driven analysis was conducted to examine the unfolding of the deviancy effect (deviant–standard) using non-parametric cluster-based permutations computed on ERP amplitudes from stimulus onset up to 600 ms. Specifically, we examined differences between signers and speakers in the deviancy effect (standard vs. deviant), separately for each standard-deviant pair (pen, chair, bar). Spatial threshold was fixed at 2 clustered electrodes, and we used 2000 randomizations. Analyses were performed using the Brainstorm software package^46^ with the function FT_timeskects^47^. Another kind of analysis focused on P300. When (as in our task) target discrimination is easy and demand of focal attention is minimal, the largest P300 amplitude is observed over central/parietal electrodes^48^. We examined the ERPs differences between standards and deviants over the Pz electrode. Mean amplitudes for standards and deviants were examined using a mixed repeated-measures ANOVA; Greenhouse-Geisser correction for non-sphericity was applied when appropriate. A final analysis, modelled after Baus and Costa^31^, examined the iconicity effects on signers’ responses to pen (iconic) and chair (non-iconic) at 70–140 ms, in two electrode clusters (Posterior: Pz, P3 and P4; Occipital: Oz, PO9 and PO10). To discard effects of deviancy, this analysis was restricted to standards.

## Supplementary information

Appendix.

## Data Availability

The datasets generated during and/or analysed during the current study are available at https://osf.io/y4pb9/?view_only=4357b4a8a46f481f93ea94a3eebd7f9c.
