# Supplementary material for: Language can shape the perception of oriented objects
Source: Sci Rep. 2020 May 21;10:8409. doi: 10.1038/s41598-020-65455-6 (PMC7242439; doi:10.1038/s41598-020-65455-6)
Supplement: Supplementary file 1 — Appendix. [file 41598_2020_65455_MOESM1_ESM.pdf]

Language can shape the perception of oriented objects

Eduardo Navarrete, Michele Miozzo & Francesca Peressotti

Appendix

Objects (n = 28) displayed by LIS signs in a specific orientation that were tested in Experiment 1: Airplane, broom, brush, carrot, celery, clothespin, dart, fish, flag, fork, guitar, iron, key, knife, leaf, match, needle, oar, pen, pliers, racket, rifle, salami, scissors, screwdriver, syringe, spoon, toothpaste.
